# The development of a survey instrument to measure the barriers to the conduct and application of research in complementary and alternative medicine: a Delphi study

**DOI:** 10.1186/s12906-018-2352-0

**Published:** 2018-12-14

**Authors:** Yasamin Veziari, Saravana Kumar, Matthew Leach

**Affiliations:** 1 School of Health Sciences, University of South Australia, North Terrace, Adelaide, SA 5000 Australia; 2 Department of Rural Health, University of South Australia, North Terrace, Adelaide, SA 5000 Australia

**Keywords:** Complementary and alternative medicine, Evidence-based practice, Research, Barriers, Delphi study, Consensus

## Abstract

**Background:**

As Complementary and Alternative medicine (CAM) grows in popularity, there is overt recognition for research evidence to inform clinical practice. While various strategies have been trialed to overcome the barriers to such progress, it is important to first understand the extent to which these barriers impact the conduct and application of CAM research. This study aimed to garner consensus and agreement among CAM researchers and practitioners in refining and validating a preliminary survey instrument for measuring these barriers.

**Methods:**

A three-round Delphi study was undertaken with 22 international experts of CAM research and practice. Round one gathered consensus on 30 statements listing barriers to the application and conduct of CAM research. Only rounds two and three sought consensus on revised statements. Any statement demonstrating ≥ 70% agreement on a four-point Likert scale was determined to have reached consensus.

**Results:**

Consensus and agreement was achieved for all 30 statements listing the barriers to the application and conduct of research in CAM. Additional commentary by the Delphi participants directed whether a statement had to be split into two parts or reworded. This process resulted in the generation of the “BarrierS To the Application and Conduct of rEsearch” (oBSTACLES) instrument.

**Conclusion:**

This study, the first of its kind on this topic, identified consensus and agreement on a series of evidence-informed statements to measure the barriers to the conduct and application of research in CAM. The uniqueness of the oBSTACLES instrument is its ability to measure barriers across the evidence-based continuum.

**Electronic supplementary material:**

The online version of this article (10.1186/s12906-018-2352-0) contains supplementary material, which is available to authorized users.

## Background

Complementary and Alternative medicine (CAM) refers to a large group of healthcare practices that are not part of a country’s own conventional medicine and are not integrated into mainstream healthcare of a country [1]. CAM therapies follow a holistic model of health with the practitioner-client relationship being an important element of practice [2]. This group of therapies employ interventions/techniques that promote the innate healing ability of the body while retaining a core focus on individuality, holism, education and disease prevention [3]. The growing use of CAM across the globe [4] can be attributed to many factors, including but not limited to, the move towards holistic well-being, the recognition of the limitations associated with conventional medicine and the increasing discourse on the important contribution of CAM [5]. As the popularity of CAM has increased, so has the level of scrutiny about the evidence underpinning its effectiveness [6]; resulting in calls for increased research and an appraisal of the evidence [7, 8]. There is a view amongst the scientific community that CAM, unlike conventional medicine, is not underpinned by rigorous research [9], and this has resulted in calls for increased research and a balanced appraisal of the evidence in order to improve confidence in the CAM industry [7].

Parallel to the calls for more CAM research is the increasing recognition of the numerous barriers to conducting rigorous research in CAM [8]. Furthermore, translation of evidence into practice in CAM continues to face challenges [10–15] that result in persistent knowledge and practice gaps [16]. While the existence of these issues in CAM research and practice are recognised, there is no single instrument that adequately measures the barriers to the conduct of research and the application of research evidence in CAM. While some measures are available that explore the barriers to the utilisation of research in practice, such as the “Barriers to Research Utilization Scale” (BARRIERS) [17] and the “Evidence-Based practice Attitude and utilization SurvEy” (EBASE) [18], these tools do not address barriers pertinent to both the *conduct* of CAM research and the *application* of research evidence in CAM practice. The former is important, as the identification of barriers to the *conduct of research* is the necessary first step towards improving the quality of research in CAM [19]; identifying the barriers to the *application of research* is a necessary second step to ensuring that the findings of such research are utilised in clinical practice.

One factor possibly contributing to the slow uptake of evidence-based practice (EBP) in CAM may be that the barriers to the conduct of research and the application of research evidence in CAM are not well understood, or alternatively, have not been adequately measured. This highlights the need for an instrument that can identify the barriers to the conduct as well as the application of research in CAM. Identifying these obstacles to CAM research conduct / application will set the path to establishing a stronger research culture, building a quality evidence base, and improving the uptake of the best available evidence in CAM practice.

This research presents the third and final stage of a multi-method project designed to develop an instrument to measure the barriers to the conduct and application of research in CAM. Stage one of the project was a systematic review, the purpose of which was to synthesise the evidence pertaining to the barriers to the conduct and application of research in CAM [20]. The barriers that were unique to the *conduct* of research were captured within one of two categories: “capacity” and “culture”. “Capacity” referred to barriers in the areas of access, competency and bias. Access related to funding, training, and skills; bias related to the inherent negative perceptions about CAM research; and competency referred to the skills, knowledge, experiences and research education of the CAM practitioner in terms of conducting research. Barriers identified within the “culture” category were broadly classified as values and complex systems. Values related to inherent practices, reluctance to engage with mainstream research, historical perspectives, and an educational model that places little emphasis on research evidence. Complex systems referred to the complexity underpinning CAM research, the inability to undertake blinding and/or use placebo controls, and the limited generalisability of findings.

The barriers to the *application* of research were similarly captured within the categories of “capacity” and “culture”. Under “capacity” were barriers pertaining to access, competency and bias. Access related to limited resources, limited quality evidence and insufficient skills. Competency referred to the limited research skills of a practitioner and the inability to interpret and impart results. Within the sub-category of bias were negative perceptions of research, historical viewpoints leading to the antithesis of EBP in CAM, the lack of incentives in CAM research and lack of time. The category of “culture” captured a number of barriers relating to beliefs, lack of interest in research and infrequent use of bibliographic databases. All of these barriers to the conduct and application of research in CAM provided the necessary framework for a provisional survey instrument. Statements to be included in the provisional survey instrument were developed using a nominal group technique.

Stage two of the research project involved conducting a nominal group technique (NGT) [21]. The NGT brought together the findings from the systematic review, as well as the expertise of local CAM providers and researchers, to develop a preliminary list of statements for a pilot survey instrument. The experts were selected using purposive sampling; this ensured that participants with pertinent expertise and personal attributes were selected [22]. The nominal group technique consisted of 7 stages [21]: In stages 1 and 2 (welcome and deliberations), an A3 document containing a list of all barriers generated from the stage 1 systematic review were handed to the participants. Adjacent to these barriers were 72 examples of potential statements that reflected these barriers. This included 44 statements relating to the barriers to the conduct of research in CAM, and 28 statements relating to the barriers to the application of research in CAM. Each participant individually considered the issues to be deliberated. For stages 3 and 4 (generation of ideas/themes and discussions), each participant disclosed the results of their deliberations. The group discussed that it had understood the items that were put forward, and that all participants agreed that each of the statements were relevant barriers to the field of CAM. Attention was then directed towards the wording of the statements, of which some statements underwent minor editorial changes. In stages 5 and 6 (evaluation and consensus), ideas were evaluated; participants agreed upon ideas; and the list of 72 examples of potential statements presented in stage 2 evolved into 36 statements. For stage 7 (data refinement), the developed ideas were reworded and rearranged, and key constructs for themes that were suggested by the NGT participants were taken into consideration in the design of the preliminary survey instrument.

The outcome of the NGT was the development of a preliminary survey instrument consisting of 30 statements, with the *conduct* section of the instrument consisting of 16 statements, and the *application* section of the instrument comprising 14 statements. This instrument was referred to as “BarrierS To the Application and Conduct of rEsearch (oBSTACLES)”. This preliminary instrument was later refined and validated through a Delphi study involving international experts in CAM. This manuscript reports the findings from this Delphi study.

### Aim

The aim of this study was to refine and validate a preliminary survey instrument for measuring the barriers to the conduct and application of research in complementary and alternative medicine.

## Methods

### Design

#### The Delphi study

A three-round Delphi study was carried out for this research. This iterative approach provided a means of reaching consensus on a complex healthcare problem [23] in order to improve policy, research and clinical decision-making [24, 25]. Accordingly, the study design was most suitable in addressing the aim of this study.

The Delphi method explores divergence in an iterative series of rounds in order to gain consensus from a panel of experts [25]. The rounds involve the presentation of an issue to a panel of experts to seek their opinion. Once all the participants respond, the data are summarised, and subject to the responses provided, a second round is sent out. First-round responses of participants are collated and analysed to form the second round and so on [26]. Repeat rounds are continued until consensus is reached [27]. Delphi studies do not have a set limit on the number of rounds to be undertaken. At the discretion of the researcher [28], two rounds [27] or more may be undertaken, with three rounds being common [29].

#### Participants

Establishing a panel of experts is a core requirement of a Delphi study [30]. For this research, the panel comprised of international experts in CAM research and practice, recruited from within three geographical areas where CAM is widely practiced and researched, namely 1) Australasia, 2) Europe and 3) North America. A description of the participant inclusion and exclusion criteria is presented in Table 1.

**Table 1:** Overview of the participant inclusion & exclusion criteria

**Inclusion criteria**		**Exclusion criteria**
Researchers	Practitioners	Researchers/Practitioners
• Researchers who have a particularistic focus in CAM research • Researchers who preferably have a PhD or a minimum of a post graduate qualification • Researchers who have published in the area of expertise • Researchers who have an appointment or affiliation with a governing organisation	• Practitioners from disciplines such as naturopathy, homeopathy, herbalism, chiropractic, massage therapy or acupuncture/TCM and osteopathy • Practitioners working in CAM for at least 10 years • Practitioners with a minimum of a post graduate qualification • Practitioners who have an appointment or affiliation with a governing organization	• Medical/health researchers who research CAM as an ancillary • Practitioners of reiki, meditation, aromatherapy or hypnosis

#### Sample size

No guidance on appropriate sample sizes for Delphi studies [31] currently exists. Instead, the scope of the problem and available resources typically determines participant numbers [32, 33]. Furthermore, larger sample sizes do not necessarily equate with better responses, with data collection and analysis increasing in complexity with increasing sample size [27]. Given the specific focus of this research, the resources available and time constraints, a sample size of 21–24 was considered adequate [34]. Hence, this study aimed to recruit 7–8 participants per geographical region, which is similar to sample sizes reported in other Delphi studies [35, 36].

#### Sampling and recruitment

The sampling strategy encompassed three approaches: 1. Stratification sampling to ensure adequate representation of each geographical region [37]; 2. Purposive sampling to enable the selection of participants based on their expertise and willingness to share their expertise [22], of which CAM experts were selected from countries where CAM is widely practiced and researched; and 3. Snowball sampling [38, 39], which identified additional CAM researchers and practitioners. The participants from stage 2 (NGT) were invited to suggest names of international or national researchers/practitioners who may have been able to contribute to this research. Additionally, where contact details could be obtained, authors of publications identified in stage 1 (systematic review) were invited to participate in this Delphi study.

Recruitment followed the Dillman Protocol [40]. An invitation email informed eligible participants about the proposed research and the importance of participating in all rounds to minimise attrition bias [41]. If there was no response to the invitation within 1 week, a second reminder was sent [42]; if there was still no response, it was assumed that the participant was unavailable, and no further attempts were made to contact the participant.

### Procedure

#### Data collection

Once the desired number of participants had responded, participants were sent an invitation email containing an active hyperlink (this process was also followed for rounds two and three). Upon entering the survey, participants provided informed consent, and then proceeded to commence round one of the Delphi study. Participants were given 4 weeks to complete each round. Reminders were sent to late respondents (after 4 weeks), which included a 2-week extension to complete the round. Those that did not respond within the six-week period were classified as non-respondents, with no further attempts made to contact them. All three Delphi rounds used the online survey platform, SurveyMonkey™ [43]. In the first round, participants were presented with all 30 statements (Table 2) from the preliminary survey instrument (i.e. derived from stage 2), with statements divided into two parts: barriers to the conduct of research (part A) and barriers to the application of research findings (part B). In rounds two and three, participants only received statements that had undergone some degree of revision (i.e. statements that had not yet been accepted or rejected by the majority of experts). All participants that participated in round one were given the opportunity to participate in rounds two and three.

**Table 2:** The Preliminary survey instrument

**Part A: Barriers to the** ***conduct*** **of research in CAM**	**Part B: Barriers to the** ***application*** **of research in CAM**
1. There are limited funding opportunities to conduct research in CAM. 2. There are limited incentives for researchers to participate in CAM. 3. There are limited opportunities for CAM practitioners to contribute to CAM research. 4. There are limited numbers of dedicated CAM researchers 5. There are limited numbers of journal/grant reviewers with expert understanding of CAM. 6. There are limited large scale and long-term CAM research studies. 7. There are limited opportunities to recruit CAM research participants due to negative public attitudes towards CAM. 8. There are negative perceptions of CAM research amongst those outside the field of CAM. 9. There are limited opportunities to publish CAM research in scientific journals. 10. There is limited collaboration between CAM and other health researchers. 11. There are limited numbers of CAM researchers who have dedicated expertise in a CAM discipline. 12. There is limited recognition for the value of research within CAM. 13. There are limited opportunities for research skills development in CAM undergraduate education. 14. There are limited opportunities for CAM undergraduate students to contribute to CAM. 15. Traditional research designs are of limited value to test complex individualised CAM treatments. 16. The reductionist biomedical model of care is not compatible with the broader CAM holistic model of care.	17. CAM practitioners have limited *access* to research evidence. 18. CAM practitioners have limited *access* to research training. 19. CAM practitioners have limited *awareness* of clinical practice guidelines. 20. CAM practitioners have limited knowledge and skills to *locate* the best available research evidence. 21. Publication bias poses a challenge for CAM practitioners *to locate* evidence. 22. CAM practitioners have limited knowledge and skills to *appraise* research evidence. 23. CAM practitioners have limited knowledge and skills to *apply* research evidence to practice. 24. Inconsistencies within CAM research evidence are an obstacle to *applying* research evidence to practice. 25. CAM practitioners have limited knowledge and skills to *communicate* research findings to their patients. 26. CAM practitioners have limited time to *apply* research evidence to their practice. 27. CAM practitioners have limited financial incentives to *use* research evidence to inform their practice. 28. CAM practitioners have little professional obligation to *use* research evidence to inform their practice. 29. CAM practitioners have diverse views on what constitutes research evidence. 30. CAM practitioners are often faced with patient expectations that are contrary to research evidence.

#### Data management and analyses

**Fig. 1:**
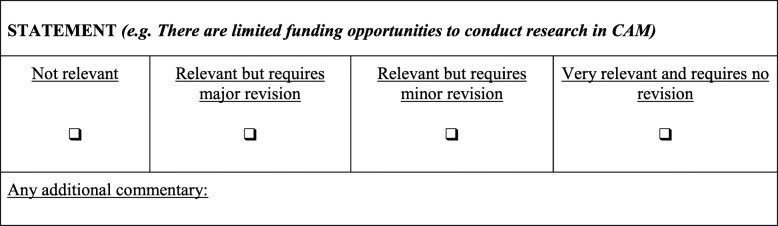
The question and response format for each statement in each Delphi round

For each round, participants were asked to review each statement and to provide a response to two questions. For the first question, *The relevance of each item*, participants were required to indicate the relevance of the proposed item on a 4-point Likert scale: category 0 = not relevant, category 1 = relevant but requires major revision, category 2 = relevant but requires minor revision, and category 3 = very relevant and requires no revision. For the second question, *Any additional commentary,* participants had to provide justification for their score, and if desired, provide additional comments. An example of these two questions is presented in Fig. 1.

Although the Delphi is a recognised technique for the investigation of opinions, it has no set agreement on consensus [23]. However, a clear definition of consensus prior to data analysis is important for credibility [33]. Therefore, as used in other health research [44–47], consensus for this study was defined as an agreement threshold ≥ 70%. Where there was ≥ 70% agreement (as described below) but qualitative comments were provided, the research team reflected on these comments and made required changes to the statements.

Agreement was expressed as the percentage of agreement to the inclusion of each statement in the instrument. Where the sum of category 2 (“relevant but requires minor revision”) and category 3 (“very relevant and requires no revision”) was ≥ 70%, the statement was included in the final instrument. If the sum of categories 2 and 3 was < 70%, and the sum of category 1 (“relevant but requires major revision”) and category 2 (“relevant but requires minor changes”) was greater than the proportion of responses to category 0 (“not relevant”), the statement was amended (based on participant comments) and added to a consecutive Delphi round to allow further mediation by the panel. An agreement rating of ≥ 50% given to category 0 (“not relevant”) meant the exclusion of the statement from the final instrument. Statements were also excluded if agreement had not been achieved within two rounds. This process was consistent with other Delphi studies [23, 48].

## Results

### Participants

**Fig. 2:**
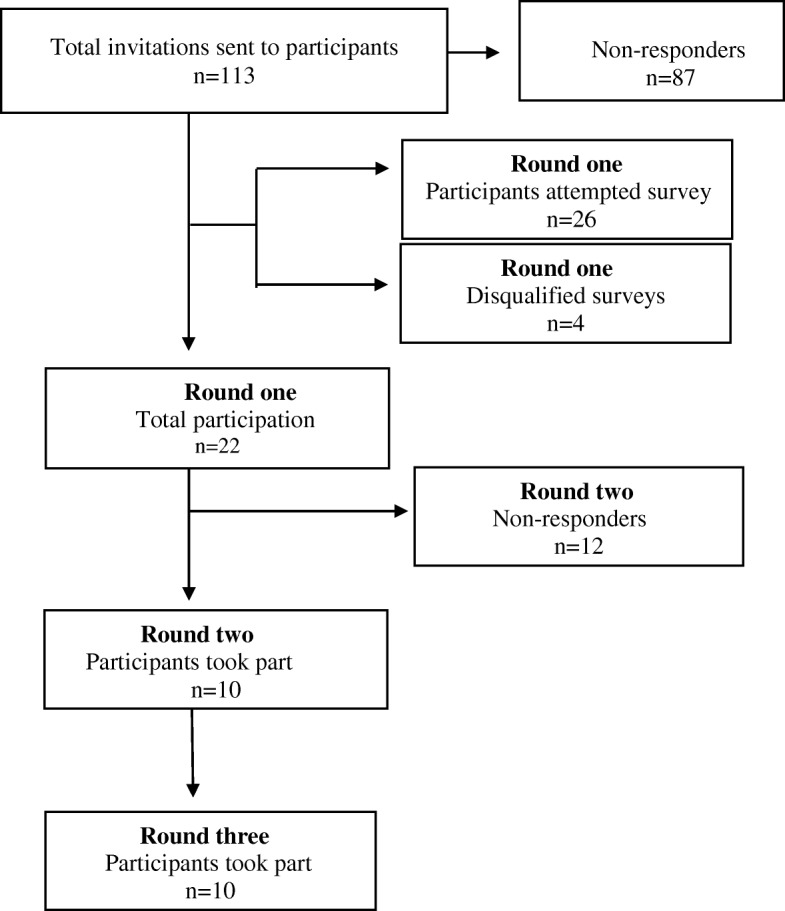
Flow of participation from round one to round three

The flow of participation from rounds one to three is presented in Fig. 2. One hundred and thirteen invitations were sent out. Round one had 26 responders, of which four incomplete surveys were excluded. Therefore, there was a total number of 22 participants with completed surveys in round one (20% response rate). Rounds two and three had 10 participants each. Over the three rounds, the same group of experts were invited to participate in this Delphi study. There was some attrition over the three rounds, which is to be expected with any research; however, the same cohort that commenced round 1 were involved in rounds 2 and 3. Overall, the participant characteristics highlight that the largest proportion of participants were aged between 40 and 49 years (31.8%, *n* = 7), with 22.7% (*n* = 5) and 18.2% (*n* = 4) aged between 50 and 59 years and 60–69 years, respectively. There were proportionally more males (63.6%, *n* = 14) than females (31.8%, *n* = 7), with one (4.5%) participant reporting other. Naturopathy was the largest professional group represented (45.5%, *n* = 10), followed by other (18.2%, *n* = 4), western herbalism (9%, *n* = 2), traditional Chinese medicine (9%, *n* = 2), homeopathy (4.5%, *n* = 1), chiropractic (4.5%, *n* = 1) and osteopathy (4.5%, *n* = 1). Over half (54.6%, *n* = 12) of participants had 15 or more years of experience in their field; 32% (*n* = 7) had 10–14 years of experience, 4.5% (*n* = 1) had 5–9 years of experience and 9% (*n* = 2) had ≤5 years of experience in their field. Most respondents (68.2%, *n* = 15) held a Doctor of Philosophy, with 13.6% (*n* = 3) holding a Professional Doctorate, 4.5% (*n* = 1) holding a Master’s degree and 4.5% (*n* = 1) holding a professional diploma; 9% (*n* = 2) were Doctor of Philosophy candidates. In terms of geographical location, ten (45.5%) participants were from Australasia, seven (31.8%) were from North America, and five (22.7%) were from Europe.

### Interpretation of the Delphi result

#### Delphi - round one

The first-round survey contained a total of 30 statements that represented the barriers to the conduct and application of research in CAM (Table 3). None of the statements in this round achieved ≥ 50% for category 0 (“not relevant”), therefore, no statements were removed. Twenty (67%) statements had reached the ≥ 70% agreement threshold for the sum of category 2 (“relevant but requires minor revision”) and category 3 (“very relevant and requires no revision”), and as such, these statements were included in the final instrument. Nine statements (30%) did not reach the agreement threshold of ≥ 70% and thus required revision.

**Table 3:**
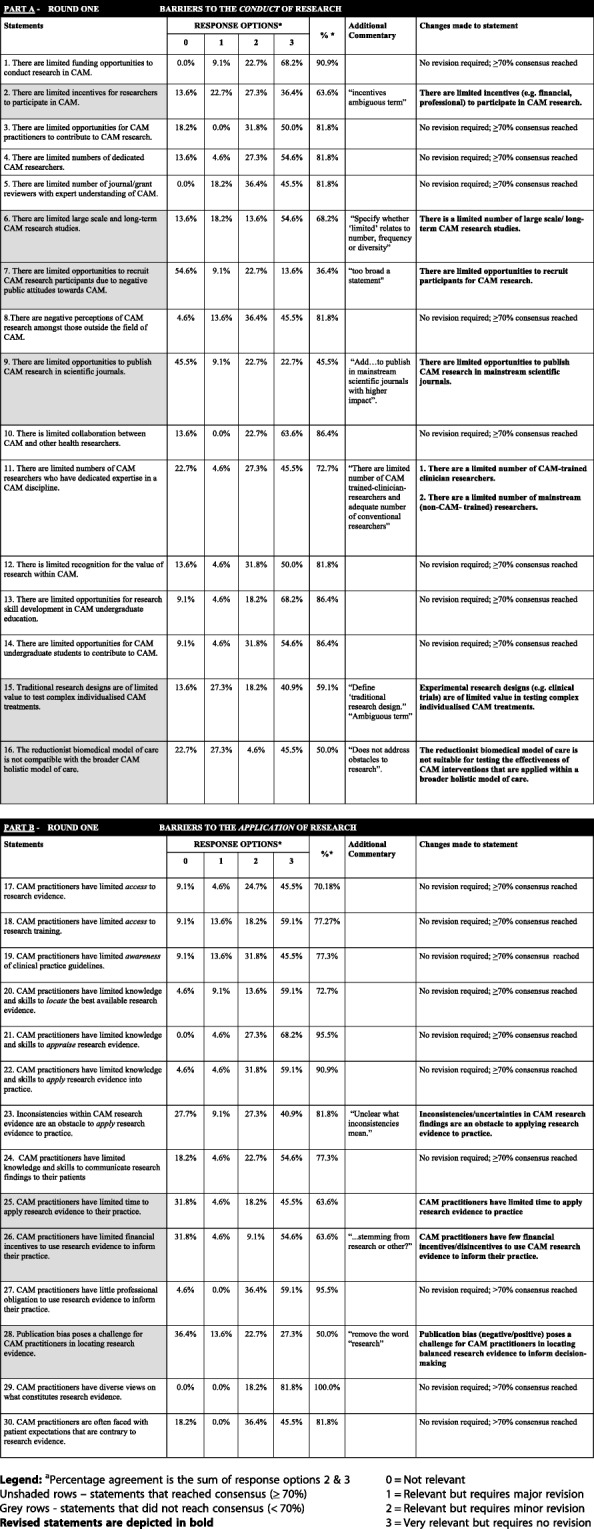
Overview of participant responses to part A and part B items from round one

Of the 20 statements added to the final instrument, those examining the barriers to the *conduct* of CAM research (Part A) included *“limited funding”* (91% agreement), *“limited opportunities to contribute to research”* (82%), *“limited dedicated researchers”* (82%), *“limited journal and grant reviewers”* (82%), *“negative perceptions of CAM from outside the field of CAM”* (82%), *“lack of collaboration between CAM and mainstream researchers”* (86%), *“limited dedicated expert CAM researchers”* (73%), *“limited recognition for the value of CAM research”* (82%), *“limited opportunities for CAM research skill development for undergraduate CAM students”* (86%), and *“limited undergraduate opportunities to contribute to research”* (86%).

The statements initially added to the final oBSTACLES instrument that examined the barriers to the *application* of research in CAM were *“limited access to research evidence”* (70% agreement), *“limited training”* (77%), *“limited awareness of clinical practice guidelines”* (77%), *“limited knowledge and skills to locate evidence”* (73%), *“appraising research evidence”* (94%), *“applying research evidence”* (91%), *“inconsistencies within CAM research evidence”* (82%), *“limited knowledge and skills to communicate research findings to their patients”* (77%), *“little professional obligation to use research evidence to inform their practice”* (95%), *“diverse views on what constitutes research evidence”* (100%), and *“patient expectations that are contrary to research evidence”* (82%).

All statements that reached ≥ 70% were accepted and not included in the subsequent Delphi rounds, except in two instances. There were a number of qualitative comments from the panel, which indicated that two statements were confusing and required further clarification; this applied to statements 11 and 23. Statement 11, *“There are limited numbers of CAM researchers who have dedicated expertise in a CAM discipline”*, had received suggestions from participants to change the wording to, “*There are a limited number of CAM trained clinician-researchers”,* and *“An adequate number of conventional researchers knowledgeable in CAM*”. Based on these comments, this statement was split into two individual statements, as follows: *“There are a limited number of CAM-trained clinician researchers”* and *“There are a limited number of mainstream (non-CAM-trained) researchers actively investigating CAM”.* Similarly, statement 23, “*Inconsistencies within CAM research evidence are an obstacle to apply research evidence to practice”,* received comments from participants indicating that it was “*unclear what inconsistencies mean*.” Therefore, the statement was changed to, *“Inconsistencies/uncertainties in CAM research findings are an obstacle to applying research evidence to practice*”. These three revised statements were included in the second Delphi round.

Nine statements (30%) did not reach the agreement threshold of ≥ 70%. These statements related to *“limited incentives for researchers to participate in CAM”* (64% agreement), *“limited large scale and long-term CAM research studies”* (68%), *“limited opportunities to recruit CAM research participants due to negative public attitudes towards CAM”* (36%), *“limited opportunities to publish CAM research in scientific journals”* (45%), *“Traditional research designs are of limited value to test complex individualised CAM treatments”* (59%), *“The reductionist biomedical model of care is not compatible with the broader CAM holistic model of care”* (50%), *“CAM practitioners have limited time to apply research evidence to their practice”* (64%), *“CAM practitioners have limited financial incentives to use research evidence to inform their practice”* (64%), and *“publication bias poses a challenge for CAM practitioners in locating research evidence”* (50%). Qualitative comments seeking changes/clarification were reviewed and considered. These nine statements were included in the round two Delphi survey (providing a total of 12 statements to be reviewed).

Table 3 provides an overview of the participant responses from round one. The “clear” rows represent statements that had reached consensus. The “grey” rows represent statements that required further refinement.

#### Delphi - round two

The response rate for the second round was 45.5% (10/22 round one respondents). None of the statements in the second round achieved ≥ 50% agreement for category 0 (“not relevant”), and thus no statements were removed from the survey. The second-round analyses determined that 11 out of the 12 (92%) statements had reached the ≥ 70% agreement threshold for the sum of category 2 (“relevant with minor changes”) and category 3 (“very relevant and requires no revision”). As such, these statements were included in the final oBSTACLES instrument. One statement (8%) did not reach the agreement threshold of ≥ 70% and thus required revision (Table 4).

**Table 4:**
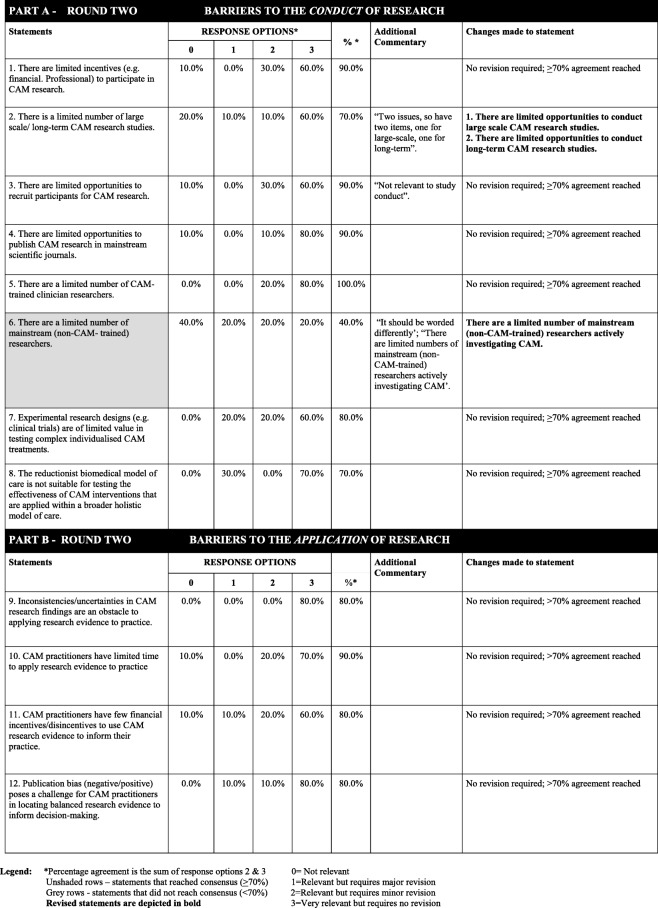
Overview of participant responses to part A and part B items from round two

The six statements from part A of the survey (barriers to the *conduct* of research in CAM) that achieved ≥ 70% agreement included *“limited incentives to participate in research”* (90% agreement), *“limited large-scale/long-term research”* (70%), *“limited participant recruitment opportunities”* (90%), *“limited CAM trained researchers”* (100%), *“experimental research designs are of limited value when testing CAM individualised treatments”* (80%), and *“the biomedical model is not suitable for testing CAM interventions”* (70%). With regards to the statement *“There is a limited number of large scale/long-term CAM research studies”,* while it achieved an agreement rating of 70%, the additional commentary from participants highlighted the following: “*Two issues, so have two items, one for large-scale, one for long-term*”. Therefore, this statement was split into two statements, as follows: *“There are limited opportunities to conduct large scale CAM research studies”* and *“There are limited opportunities to conduct long-term CAM research studies”*. These two statements were subsequently added to round three.

Statement 6 of part A, *“There are a limited number of mainstream (non-CAM trained) researchers”,* was the only statement in round two that did not achieve a ≥ 70% agreement rating (reaching only 40% agreement). Some participants suggested, *“It should be worded differently*”, and it should specify “*researchers actively investigating CAM*”. Therefore, this statement was changed to “*There are a limited number of mainstream (non-CAM-trained) researchers actively investigating CAM*” and was subsequently added to the round three Delphi survey.

The four statements from part B of the instrument (barriers to the *application* of research *in CAM*) that achieved an agreement rating ≥ 70% included *“inconsistent research findings are an obstacle to applying evidence to practice”* (80% agreement), *“limited time to apply evidence”* (90%), *“few financial incentives to use evidence”* (80%) and *“publication bias”* (80%).

A total of three statements (three from part A and zero from part B) were included in the third and final Delphi survey. Table 4 provides an overview of the participant responses from round two.

#### Delphi - round three

The response rate for the third round was 100% (10/10 round two respondents). All three statements in round three were successful in reaching the ≥ 70% agreement threshold (Table 5). These statements referred to *“limited opportunities to conducting large-scale research”* (90% agreement), *“limited opportunities to conduct long-term research”* (90%) and *“limited mainstream researchers actively investigating CAM”* (70%). On completion of the third Delphi round, 32 barrier statements that had achieved an agreement rating of ≥ 70%, from all three rounds, were incorporated into the final instrument - the BarrierS To the Application and Conduct of rEsearch (oBSTACLES) (Additional file 1). Table 5 provides an overview of the participant responses from round three.

**Table 5:**
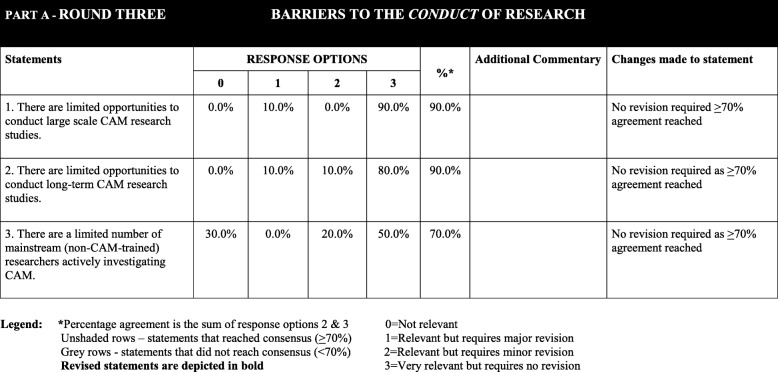
Overview of participant responses to part A items from round 3

## Discussion

Despite the increasing focus on CAM to prove its evidence-base, and growing recognition of the numerous barriers to the conduct and application of research within CAM, there has not been a single instrument that has adequately captured and measured these barriers to date. Therefore, the extent to which stakeholders of CAM are impacted by barriers to the conduct AND application of research is largely unknown. While there are generic instruments that explore CAM stakeholder’s perspectives of issues such as EBP, these instruments do not capture the range of barriers impacting through the EBP continuum (generation – access – application of research evidence). Therefore, the aim of this research was to refine and validate a dedicated instrument (oBSTACLES instrument) for measuring the barriers to the *conduct* and *application* of research in CAM.

Using the Delphi method, the panel of international experts in CAM agreed that myriad barriers exist to the conduct and application of research in CAM. The views of the experts are congruent with and supported by contemporary literature [20]. For example, the expert panel concurred that time was an important barrier to research application, and this was consistent with previous reports [10, 12, 13, 49, 50]. Also supported by previous literature was the view that access to funding was a critical barrier to research conduct [51–56]. Furthermore, the panel of international experts did not reject any statement that had formed the preliminary oBSTACLES instrument (which were developed from the systematic review and NGT). This highlights the consistency of, and consensus amongst, experts on the recognition of myriad barriers encountered when engaging with research.

The resultant output of this research is the oBSTACLES instrument. This self-administered questionnaire comprises 40 items, divided into three parts: Part A (barriers to the conduct of research in CAM; 18 statements), Part B (barriers to the application of research in CAM; 14 statements) and Part C (demographic details; 8 items). All items in parts A and B used a Likert scale response format. The oBSTACLES instrument has familiarity with other survey instruments, such as EBASE and the BARRIERS scale. The BARRIERS [17] scale, for instance, sets out to measure the factors that hinder the *application* of nursing research, taking into account the researcher, the organisation, and the innovation. EBASE [18], on the other hand, although related to CAM, investigates the factors impacting the clinical *application* of evidence-based practice, such as practitioner attitude, skills and training. However, where the oBSTACLES instrument differs from these two instruments is its focus on measuring the barriers to both the *conduct* and *application* of research in CAM. This is important, as these barriers may occur throughout the EBP continuum (generation – access – application of research evidence). It is important to capture these barriers throughout the continuum as previous research indicates that access to research alone is not sufficient [57] when promoting EBP; the focus should also be on the application of research [58]. Without such overt focus across the EBP continuum, evidence-practice gaps will continue to persist [59].

The oBSTACLES instrument also acknowledges that effective engagement with CAM research requires a contribution from more than one stakeholder [60]. In fact, the instrument draws attention to the influential role of at least six different stakeholder groups that impact EBP implementation, including researchers, educators, funders, editors/publishers, practitioners and industry (i.e. professional associations). Therefore, the data generated from using the oBSTACLES instrument may shed light on the development of strategies that are more targeted and meaningful to specific stakeholder groups, which in turn, may assist in breaking down barriers to the conduct and application of research in CAM.

As with any research, there are some limitations to this study. First, given the nature of the Delphi study methodology, there were three distinct rounds. This “stop-start-stop” process did not allow for free-flowing exchange of ideas and discussions [61]. However, the use of Delphi study methodology as means of gaining consensus on a given topic [62] is well recognised and commonly used in survey development. Second, despite considerable efforts to ensure wide-spread representation across the globe, participants from Asia and Europe were under-represented. Acquiring equal numbers of CAM practitioners and researchers also could not be controlled due to the voluntary nature of the Delphi recruitment process. As this research brought together an international sample of CAM experts from multiple geographical locations, the impact of under-representation was minimised. Third, anonymity due to privacy and confidentiality reasons may have led to a paucity of accountability [63]. Prior to the commencement of the Delphi study, the research team did request all participants to actively and openly contribute to the research. Even though this research provides evidence of consensus on the barriers to the conduct and application of research in CAM, as it used a Delphi study methodology, the findings are considered expert opinion only. Notwithstanding, as this Delphi study included CAM experts from across several other countries, the research arguably captured broad and diverse perspectives and experiences. Furthermore, as the oBSTACLES instrument has its foundation in a systematic review of the literature as well as a nominal group technique, the impact of these limitations has been minimized. Lastly, even though the oBSTACLES instrument may have potential application to areas other than CAM (such as medicine, nursing and allied health), further psychometric testing of the oBSTACLES instrument would be required.

## Conclusion

While there has been widespread recognition of the barriers that confront CAM stakeholders when engaging with research, there has been limited research that has systematically identified and measured what these barriers are across the EBP continuum. This research, by bringing together CAM experts from across the globe, has generated a series of evidence-informed statements, in the form of a survey instrument, to identify, measure and evaluate barriers to the conduct and application of research in CAM. The outcome of this research was the development of the BarrierS to the Application and Conduct of rEsearch (the oBSTACLES instrument) in CAM - a bespoke instrument that can measure and quantify barriers to both the conduct and the application of research in CAM.

### Implications for practice

The oBSTACLES instrument is a simple, easy to use instrument, which is evidence-informed as it has been developed in a methodological manner and underpinned by rigorous research processes (the systematic review, the nominal group technique and the Delphi study). It builds on existing tools such as EBASE and the BARRIERS scale, while addressing their limitations. This instrument can be applied across a range of different practice settings spanning the spectrum of CAM disciplines. As the oBSTACLES instrument measures and quantifies barriers to the conduct and application of research in CAM, it will assist in the development of targeted enabling strategies to overcome these barriers. By doing so, the oBSTACLES instrument provides opportunities to address entrenched barriers that confront CAM stakeholders when engaging with research.

### Implications for research

While the development process for the oBSTACLES instrument was underpinned by a systematic and rigorous methodology, formal psychometric testing remains to be undertaken. Areas of future research include establishing reliability, validity and utility of the oBSTACLES instrument, as well as formal testing with other health professional groups (such as nursing and allied health). With increasing focus on not just the generation of evidence but also on the implementation and evaluation of evidence in health care, the oBSTACLES instrument provides ideal opportunities for future research to examine, measure and understand those barriers that exist not in isolation but across the evidence-based continuum. In turn, the instrument may help to determine what enabling strategies may work best for whom and under what contexts.

## Additional file

Additional file 1: The oBSTACLES instrument. (DOCX 43 kb)

## Abbreviations

CAM: Complementary and alternative medicine
EBP: Evidence-based practice
oBSTACLES instrument: BarrierS To the Application and Conduct of rEsearch instrument
