# Supplementary material for: The development of a survey instrument to measure the barriers to the conduct and application of research in complementary and alternative medicine: a Delphi study
Source: BMC Complement Altern Med. 2018 Dec 14;18:335. doi: 10.1186/s12906-018-2352-0 (PMC6295050; doi:10.1186/s12906-018-2352-0)
Supplement: Supplementary file 1 — The oBSTACLES instrument. (DOCX 43 kb) [file 12906_2018_2352_MOESM1_ESM.docx]

**The oBSTACLES Instrument**

**______________________________________**

***BarrierS To the Application and Conduct of rEsearch***

Thank you for agreeing to take part in this important survey. The oBSTACLES instrument captures the barriers to the conduct (Part A) and the application (Part B) of research in Complementary and alternative medicine (CAM). For each of the statements listed in Parts A and B of the instrument, please indicate the extent to which you agree with the statement, ranging from ‘strongly disagree’ to ‘strongly agree’. Please avoid selecting ‘uncertain’ unless you are truly unsure. Thank you again for your participation.

**PART A: BARRIERS TO THE *CONDUCT* OF RESEARCH**

| *Please indicate your opinion of each barrier by ticking one box only for each statement.* | | **Strongly disagree** | **Disagree** | **Uncertain** | **Agree** | **Strongly agree** |
| --- | --- | --- | --- | --- | --- | --- |
| 1. | There are limited funding opportunities to conduct research in CAM. | ❑ | ❑ | ❑ | ❑ | ❑ |
| 2. | There are limited incentives (e.g. financial, professional) to participate in CAM research. | ❑ | ❑ | ❑ | ❑ | ❑ |
| 3. | There are limited opportunities for CAM practitioners to contribute to CAM research. | ❑ | ❑ | ❑ | ❑ | ❑ |
| 4. | There are limited numbers of dedicated CAM researchers. | ❑ | ❑ | ❑ | ❑ | ❑ |
| 5. | There are a limited number of journal / grant reviewers with expert understanding of CAM. | ❑ | ❑ | ❑ | ❑ | ❑ |
| 6. | There are limited opportunities to conduct large scale CAM research studies. | ❑ | ❑ | ❑ | ❑ | ❑ |
| 7. | There are limited opportunities to conduct long-term CAM research studies. | ❑ | ❑ | ❑ | ❑ | ❑ |
| 8. | There are limited opportunities to recruit participants for CAM research. | ❑ | ❑ | ❑ | ❑ | ❑ |
| 9. | There are negative perceptions of CAM research amongst those outside the field of CAM. | ❑ | ❑ | ❑ | ❑ | ❑ |
| 10. | There are limited opportunities to publish CAM research in mainstream scientific journals. | ❑ | ❑ | ❑ | ❑ | ❑ |
| 11. | There is limited collaboration between CAM researchers and other health researchers. | ❑ | ❑ | ❑ | ❑ | ❑ |
| 12. | There are a limited number of CAM-trained clinician researchers. | ❑ | ❑ | ❑ | ❑ | ❑ |
| 13. | There are a limited number of mainstream (non-CAM-trained) researchers actively investigating CAM. | ❑ | ❑ | ❑ | ❑ | ❑ |
| 14. | There is limited recognition for the value of research within CAM. | ❑ | ❑ | ❑ | ❑ | ❑ |
| 15. | There are limited opportunities for research skill development in CAM undergraduate education. | ❑ | ❑ | ❑ | ❑ | ❑ |
| 16. | There are limited opportunities for CAM undergraduate students to contribute to CAM research. | ❑ | ❑ | ❑ | ❑ | ❑ |
| 17. | Experimental research designs (e.g. clinical trials) are of limited value in testing complex individualised CAM treatments. | ❑ | ❑ | ❑ | ❑ | ❑ |
| 18. | The reductionist biomedical model of care is not suitable for testing the effectiveness of CAM interventions that are applied within a broader holistic model of care. | ❑ | ❑ | ❑ | ❑ | ❑ |

**PART B: BARRIERS TO THE *APPLICATION* OF RESEARCH**

| *Please indicate your opinion of each barrier by ticking one box only for each statement.* | | **Strongly disagree** | **Disagree** | **Uncertain** | **Agree** | **Strongly agree** |
| --- | --- | --- | --- | --- | --- | --- |
| 1. | CAM practitioners have limited *access* to research evidence. | ❑ | ❑ | ❑ | ❑ | ❑ |
| 2. | CAM practitioners have limited *access* to research training. | ❑ | ❑ | ❑ | ❑ | ❑ |
| 3. | CAM practitioners have limited *awareness* of clinical practice guidelines. | ❑ | ❑ | ❑ | ❑ | ❑ |
| 4. | CAM practitioners have limited knowledge and skills to *locate* the best available research evidence. | ❑ | ❑ | ❑ | ❑ | ❑ |
| 5. | CAM practitioners have limited knowledge and skills to *appraise* research evidence. | ❑ | ❑ | ❑ | ❑ | ❑ |
| 6. | CAM practitioners have limited knowledge and skills to *apply* research evidence into practice. | ❑ | ❑ | ❑ | ❑ | ❑ |
| 7. | Inconsistencies/uncertainties in CAM research findings are an obstacle to applying research evidence to practice. | ❑ | ❑ | ❑ | ❑ | ❑ |
| 8. | CAM practitioners have limited knowledge and skills to *communicate* research findings to their patients. | ❑ | ❑ | ❑ | ❑ | ❑ |
| 9. | CAM practitioners have limited time to apply research evidence to practice. | ❑ | ❑ | ❑ | ❑ | ❑ |
| 10. | CAM practitioners have few financial incentives/disincentives to use CAM research evidence to inform their practice. | ❑ | ❑ | ❑ | ❑ | ❑ |
| 11. | CAM practitioners have little professional obligation to *use* research evidence to inform their practice. | ❑ | ❑ | ❑ | ❑ | ❑ |
| 12. | Publication bias (negative/positive) poses a challenge for CAM practitioners in locating balanced research evidence to inform decision-making. | ❑ | ❑ | ❑ | ❑ | ❑ |
| 13. | CAM practitioners have diverse views on what constitutes research evidence. | ❑ | ❑ | ❑ | ❑ | ❑ |
| 14. | CAM practitioners are often faced with patient expectations that are contrary to research evidence. | ❑ | ❑ | ❑ | ❑ | ❑ |

**PART C: DEMOGRAPHICS**

| What is your age group? |
| --- |
| ❑ Less than 19 years ❑ 50-59 years  ❑20-29 years ❑ 60-69 years  ❑ 30-39 years ❑ 70 years or over  ❑40-49 years |
| What is your gender? |
| ❑ Male  ❑ Female  ❑ Other |
| What country do you currently reside in? |
| ……………………… |
| Which **single** CAM discipline would best represent your area of CAM expertise? |
| ❑ Naturopathy ❑ Homeopathy  ❑ Chiropractic ❑ Osteopathy  ❑ Western herbalism ❑ Acupuncture  ❑ Other ………………………… |
| What are the total number of years you have worked in the above field of CAM, in any capacity (i.e. as a clinician, researcher, educator, etc.)? |
| ❑ Less than 5 years ❑ 10-14 years  ❑ 5-9 years ❑ 15 years or more |
| How many hours do you spend performing the following roles in a typical working week? |
| Clinician: …………................ Hours Educator: ……………. Hours  Researcher: ………................. Hours Administrator: ………. Hours  Other (please specify): ……… Hours |
| What is your highest level of education? |
| ❑ Certificate ❑ Graduate certificate/diploma  ❑ Diploma ❑ Master’s Degree  ❑ Advanced Diploma ❑ Professional doctorate  ❑ Bachelor degree ❑ Doctor of Philosophy (PhD)  ❑ Honours degree ❑ Other (please state)  …………………………………. |
| Is there any other additional information regarding this particular topic that you would like to make? |
|  |
| *Thank you for your valuable time and input in this survey* |
| For more information:  Yasamin Veziari  School of Health Sciences  University of South Australia  Telephone: 61 412950891  E mail: [Yasamin.Veziari@mymail.unisa.edu.au](mailto:Yasamin.Veziari@mymail.unisa.edu.au)  Dr Saravana Kumar  Senior Lecturer  School of Health Sciences  University of South Australia  Telephone: 61 (08) 8302 2085  E mail: [Saravana.Kumar@unisa.edu.au](mailto:Saravana.Kumar@unisa.edu.au)  Dr Matthew Leach  Senior Research Fellow   \| **Version:** \| V1 \| \| --- \| --- \| \| **Date of version** \| October 2018 \| \| **Delegated Authority:** \| Yasamin Veziari  Dr Matthew Leach  Dr Saravana Kumar \|   Department of Rural Health  University of South Australia  Telephone: 61 (08) 8302 2413  Email: [Matthew.Leach@unisa.edu.au](mailto:Matthew.Leach@unisa.edu.au)    The OBSTACLES instrument was developed as a result of an Honours research by Yasamin Veziari (honours student) and supervisors, Dr Matthew Leach & Dr Saravana Kumar at the University of South Australia. The oBSTACLES instrument is free to use in its original form. The end user(s) shall not modify, abridge, condense, adapt, recast, or transform the oBSTACLES instrument in any manner or form, without the prior written agreement of the developers. This includes, but is not limited to, any change to the words and/or the organisation of the questions contained in the oBSTACLES instrument. Please contact the developers if you intend on changing the oBSTACLES instrument from its original form.  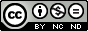  [www.augoal.gove.au/creative-commons](http://www.augoal.gove.au/creative-commons)  All rights reserved.  Printed October 2018. |
